# Management of common mental disorders should take place in primary health or specialized care? Clinical decisions of psychiatrists from Latin American countries

**DOI:** 10.1371/journal.pone.0265308

**Published:** 2022-04-05

**Authors:** Michel Haddad, Angel O. Rojas Vistorte, Glenda Guerra Haddad, Wagner Ribeiro, Carolina Ziebold, Elson Asevedo, Sara Evans-Lacko, Oscar Ulloa, Jair de Jesus Mari

**Affiliations:** 1 Department of Psychiatry, Universidade Federal de São Paulo, São Paulo, Brazil; 2 Institute of Psychiatry, Hospital das Clínicas, FMUSP, São Paulo, Brazil; 3 London School of Economics and Political Science – Care Policy and Evaluation Centre, London, United Kingdom; 4 Global Mental Health Program, Columbia University, New York, New York, United States of America; 5 King’s College London, Health Service and Population Research Department, Institute of Psychiatry, Psychology & Neuroscience, London, United Kingdom; 6 Rioja University, Logrono, Spain; Assistance Publique Hopitaux de Marseille, FRANCE

## Abstract

**Objective:**

The objective of our study was to explore clinical decisions of psychiatrists regarding the management of common mental disorders in primary care (PC) in four Latin Americans countries, through the application of clinical vignettes.

**Methods:**

Using a cross-sectional design, we conducted a self-administered online questionnaire survey of psychiatrists from Bolivia, Brazil, Cuba, and Chile. The questionnaire covered sociodemographic and professional information. The psychiatrists’ clinical decisions were assessed through three clinical vignettes representing typical PC cases of depression, anxiety, and somatization.

**Results:**

230 psychiatrists completed the online survey. Psychiatrists from Brazil were less likely to recognize depression as a mental disorder than those from Cuba (odds ratio (OR) = 0.30, 95% confidence interval (CI), 0.10 to 0.91, *p* < 0.04). Female gender (OR = 0.19, 95% CI, 0.04 to 0.91, *p* < 0.02) and older age (OR = 0.92, 95% CI, 0.87 to 0.97, *p* < 0.01) reduced the likelihood of agreement that depression cases should be treated by a Primary Care Physician (PCP). In the somatoform symptoms vignette, longer training duration increased the likelihood of agreement that treatment should be done by a psychiatrist instead of a PCP (OR = 1.19, 95% CI, 1.04 to 1.37, *p* < 0.01). In the anxiety vignette, females (OR = 2.38, 95% CI, 1.10 to 5.13, *p* < 0.01) and participants from Bolivia (compared with Cubans, OR = 4.19, 95% CI, 1.22 to 14.42, *p* < 0.02) were more likely to consider that these patients should be treated by a psychiatrist instead of a PCP.

**Discussion:**

Most psychiatrist respondents agreed that patients with depression should be treated by PCPs and that somatoform and anxiety cases should be treated by psychiatrists. These results show that psychiatrists consider that they, and not PCPs, should treat patients with common mental disorders, regardless of the evidence showing that common mental disorders can be treated by primary care physicians in PC.

## Introduction

In primary care (PC) settings, the prevalence of mental health disorders is high. Studies estimate that 24% of patients who visit primary care physicians (PCPs) have a well-defined ICD-10 mental disorder [1]. Wang et al. [2] pointed out that 90.4% of individuals who reported depressive episodes had contact with general practitioners in the previous 12 months. These data indicate the importance of PC in the management of mental disorders, especially in countries with fewer medical specialists.

The prevalence of mental disorders generates a demand for mental health care that is disproportionate to the number of specialists available [3]. In Latin America for example the prevalence of mental disorders in 2017 was 12.9%, and specifically in the countries of our study was 15.2% in Brazil, 12.4% in Bolivia, 16.5% in Chile, and 14.9% in Cuba. Thus, it has been proposed that most people with mental disorders, particularly those with common mental disorders, should be treated in primary health care (PC) [4, 5]. Evidence shows that non-mental health specialists provide more than 90% of mental health care worldwide [3, 6]. Therefore, integrating mental health into PC is a global priority. In 2013, the World Health Assembly adopted the World Health Organization (WHO)’s Comprehensive Mental Health Action Plan 2013–2020, one of whose objectives is the provision of “comprehensive, integrated, and responsive mental health and social care services in community-based settings,” principally in response to the high prevalence of common mental disorders, such as depression and anxiety [7]. Early studies examining the care provided to such patients found that Primary Care Physicians (PCPs) frequently failed to recognize psychiatric disorders, over- or under-prescribed psychotropic medications, rarely provided structured counseling, and infrequently referred patients for mental health services [8, 9].

In a systematic review of 63 studies on strategies to increase the availability of human resources for mental health care, Kakuma et al. note that PCPs who receive mental health training can efficiently identify, diagnose, and treat mental disorders [5]. The authors also suggest that if PCPs are engaged in the treatment of common mental disorders, the limited number of available mental health specialists can focus on more complex clinical situations [5]. However, there are barriers that hinder PCPs’ ability to properly treat mental disorders and contribute to an increased probability of these patients being referred to mental health specialists. First, despite the high prevalence of psychiatric disorders in PC populations, only 5.4% of patients seen by PCPs present with a psychiatric disorder as the main complaint [10, 11]. Second, a lack of training throughout medical education, from undergraduate courses to specialization, makes it difficult for PCPs to identify mental disorders [12]. In addition to limiting the capacity for management in this population, the lack of training contributes to the prevalence of misconceptions about mental disorders [10, 11]. Even in developed countries such as the Netherlands, most patients with depression (57.9%) attended by general practitioners were referred to specialized mental health services.

Despite many trials reporting successful models of collaborative care that commonly involved a PCP, a mental health professional, and a case manager, the reality shows that much of the time the participating mental health professionals were psychiatric nurses, psychologists, or social workers instead of psychiatrists [13–15]. Also, some psychiatrists perceived a lack of time and remuneration to sustain their regular visits to primary care settings [16, 17]. Recent studies report a lack of collaboration and consensus on protocol of care between PCPs and psychiatrists [12, 13]. This can lead to significant barriers to the integration and provision of mental healthcare services. It is therefore important to understand the views of the PCPs and psychiatrists, who are the key players of mental health care provisions [18]. In a recent study, public and private psychiatrists supported the management of common mental health problems by PCPs if there was appropriate collaboration and understanding of their limitations. Some of them pointed out that the decision and timing for referral should be flexible [18]. Many studies have examined the factors that lead PCPs to treat or refer patients with depression to mental health specialists [19, 20], but little is known about psychiatrists’ perceptions of and clinical decisions regarding the treatment of common mental disorders in PC.

The objective of our study was to explore clinical decisions of psychiatrists regarding the management of common mental disorders in primary care (PC) in four Latin Americans countries, through the application of clinical vignettes.

Each country of our sample presents important differences concerning the proportion of mental health professionals, and specifically psychiatrists for the care of common mental disorders, and in the case of our study, we found Cuba, with the highest rate in Cuba, with 11 psychiatrists per 100.000 inhabitants, and the lowest rate in Bolivia, with 1.30 psychiatrists per 100.000 inhabitants [21, 22]. Also, all the countries, and specifically the four of our study, have important differences in their mental health policies, and their socio-economics systems, which may impact on the responses of psychiatrists in the carried-out study.

## Method

### Sample

A convenience sample was recruited by members of our team according to the following criteria: Psychiatrists habilitated to and working as clinical psychiatrists in Bolivia, Brazil, Cuba, or Chile. These countries were selected because they represent different levels of economic development and mental health services organizations and policies. We obtained a list of psychiatrists from the four countries from the members of our team through their contacts in the health systems. We invited 380 psychiatrists from Bolivia (La Paz), Brazil (whole country), Cuba (whole country), and Chile (Santiago) to participate in the study.

### Instruments

Data collection was carried out between April and July 2016 through an online survey using the *Qualtrics* platform, sending the invitation to participate by e-mail [23], which facilitates the administration of questionnaires in a simple format. In Cuba, since access to the Internet is very limited, questionnaires were applied in print versions in the care centers where psychiatrists worked.

The questionnaire was designed to obtain information related to professionals’ profiles, including years of professional experience working as a psychiatrist, years of formal medical training, level of knowledge in different aspects of mental disorders, the diagnostic systems in which they were trained (e.g., DSM-V, ICD-10), and whether these classifications are useful to them in diagnosing and treating mental health problems in PC.

Intended clinical decisions regarding mental health problems were evaluated using three clinical vignettes describing patients with symptoms of depression (V1), somatization (V2), and anxiety (V3) according to the ICD-10 clinical descriptions and diagnostic guidelines (CDDG) [24]. The clinical vignettes were developed by two psychiatrists from our team with extensive clinical experience, as well as two general practitioners working in the field of mental health. The vignettes described typical symptoms and/or complaints of patients with common mental disorders seeking treatment in PC. Following each vignette, we asked psychiatrists to evaluate whether patients described in the vignettes: 1) presented mental health symptoms that would require care/treatment, 2) should be treated in PC, 3) should be referred to specialized mental health care, and 4) should be prescribed psychotropic medicine. Each question was scored on a four-point Likert scale, where 1 = *strongly agree*, 2 = *partially agree*, 3 = *partially disagree*, and 4 = *strongly disagree*. Participants had to choose one of the four possible alternatives for each of the possible clinical decisions for each case. Two of the three vignettes were randomly presented to each psychiatrist.

### Data analysis

Data were analyzed using SPSS for Windows version 22. First, we calculated frequency distributions for categorical variables, as well as means and standard deviations for continuous variables. The primary outcomes were the psychiatrists’ intended clinical decisions. In order to facilitate the statistical analysis, responses to the clinical vignettes were dichotomized into *yes* (*strongly agree* and *partially agree*) or *no* (*strongly disagree* and *partially disagree*) to create four variables assessing whether participants believed that patients described: 1) had a mental disorder, 2) should be treated in a PC, 3) should be recommended psychiatric treatment, and 4) should be prescribed an antidepressant. We assessed the association of clinical decision variables with sociodemographic and professional characteristics (age, gender, training duration, etc.) through bivariate associations. We used 2×2 contingency tables when variables were categorical, Student’s *t*-tests when variables were continuous, ANOVA, when the independent variable had more than two categories (nationality, for example), and non-parametric tests if the sample did not have a normal distribution. Then, using generalized linear models (GLM), we assessed the associations between clinical decisions (dichotomous responses to the clinical vignettes, with binomial distributions, as explained above) with sociodemographic factors (gender, nationality) and sociodemographic and professional covariates (age and years of training). For all statistical tests, we adopted a 5% significance level (*p* < 0.05) and 95% confidence interval (CI).

### Ethics statements

The study design was reviewed and approved by the Ethics Committee of the Federal University of Sao Paulo (UNIFESP) (CEP Project/UNIFESP 0259/2018 Number: 84901418.1.0000.5505). Participants received detailed information about the research project and were enrolled only after they had provided written informed consent. To guarantee anonymity, participants were de-identified in the database.

## Results

### Sample characteristics

A total of 230 psychiatrists filled out the online questionnaires (102 from Brazil, 29 from Bolivia, 29 from Chile, and 70 from Cuba) (65.7% response rate); 109 (47.3%) were female and 121 (52.6%) were male. The mean age of the sample was 44.5 years (standard deviation (*SD*) = 9.5), ranging between 29 and 78 years. Mean formal training duration was 9.4 years (*SD* = 6.2). There was no significant difference in ages of the participants between countries (*F*(3) = 2.09; *p* = .10). Cuban and Chilean psychiatrists had more years of training (*F*(3) = 5.9; *p* = .01) than those from Bolivia and Brazil (Table 1).

**Table 1:** Demographics and training characteristics of the participating psychiatrists.

		Bolivia	Brazil	Chile	Cuba	p-value*
		(N = 29)%	(N = 102) %	(N = 29) %	(N = 70) %	
Gender	**Female**	12(41.4%)	50(49%)	15(51.7%)	32(45.7%)	.82*
	**Male**	17(58.6%)	52(51%)	14(48.3%)	38(54.3%)	
ICD		23(79.3%)	65 (63.7%)	29(100%)	62(88.5%)	.01*
DSM		26(89.6%)	97(95%)	29(100%)	64(91.4%)	.03*
None		-	1(0.98%)	1(3.45%)	3(4.29%)	.45*
Age (Mean/SD)		42.9(10.4)	44.7(8.7)	48.2(9.2)	43.3(10.4)	.08**
Years of training (Mean/SD)		5.9(2.98)	8.80(5.50)	10.9(4.86)	11.3(7.85)	.01**
		**N(% Total)**	**N(% Total)**	**N(% Total)**	**N(% Total)**	
		**Yes**	**Yes**	**Yes**	**Yes**	
Work in Primary Care		10(34.4%)	10(9.8%)	23(79.3%)	43(61.4%)	.01*

Notes:

SD = standard deviation.

* Chi-square.

** ANOVA.

A total of 37.4% of the psychiatrists worked in PC at the moment they filled out the questionnaire; there were more Cuban (61.4%) and Chilean (79.3%) psychiatrists working in this setting than Brazilians (9.8%) and Bolivians (34.4%).

### Clinical vignettes

Most of the psychiatrists (70%) identified the presence of a mental disorder in depression (83.6%) and somatoform (84.4%) vignettes. Regarding the depression and anxiety vignettes, most of the psychiatrists from the four countries agreed in principle that these patients could be treated by a PCPs. In the depression vignette, psychiatrists from Brazil were the least likely to recognize a mental disorder (*χ*^2^(3) = 10, *p* < 0.01). In the somatoform (*χ*^2^(3) = 23.1, *p* < 0.01) and anxiety vignettes (*χ*^2^(3) = 10.6, *p* < 0.01), professionals from Brazil and Bolivia were more likely to consider that these patients should be treated by psychiatrists than professionals from Cuba and Chile. Psychiatrists from Brazil were also less likely to agree that the patients with somatoform symptoms could be treated by a PCP (*χ*^2^(3) = 11.9, *p* < 0.01) (Table 2).

**Table 2:** Clinicians’ recognition of mental disorders and decision making in the clinical vignettes, stratified by country.

		Bolivia		Brazil		Chile		Cuba		p-value (x^2^)
		n (%)		n (%)		n (%)		n (%)		
		Yes	No	Yes	No	Yes	No	Yes	No	
Depression	*Recognized a mental disorder*	13 (86.7%)	2 (13.3%)	41 (66.1%)	21 (33.9%)	19 (95%)	1 (5%)	33 (86.4%)	6 (15.4%)	.01
	*Recommended psychiatric treatment*	6 (40%)	9 (60%)	32 (57.1%)	24 (42.9%)	7 (35%)	13 (65%)	18 (46.2%)	21 (53.8%)	.29
	*Recommended treatment in PC*	15 (100%)	-	46 (82.1%)	10 (17.9%)	17 (85%)	3 (15%)	36 (92.3%)	3 (7.7%)	.20
	*Antidepressant prescription*	15 (100%)	-	46 (82.1%)	10 (17.9%)	15 (75%)	5 (25%)	29 (74.4%)	10 (25.6%)	.17
Somatoform	*Recognized a mental disorder*	16 (84.2%)	3 (15.8%)	52 (78.8%)	14 (21.2%)	13 (86.7%)	2 (13.3%)	36 (87.8%)	5 (12.2%)	.65
	*Recommended psychiatric treatment*	19 (100%)	-	59 (85.5%)	10 (14.5%)	7 (46.7%)	8 (53.3%)	24 (58.5%)	17 (41.5%)	.01
	*Recommended treatment in PC*	16 (84.2%)	3 (15.8%)	40 (58%)	29 (42%)	14 (93.3%)	1 (6.7%)	32 (78%)	9 (22%)	.01
	*Antidepressant prescription*	19 (100%)	-	66 (95.7%)	3 (4.3%)	15 (100%)	-	40 (97.6%)	1 (2.4%)	.65
Anxiety	*Recommended psychiatric treatment*	15 (75%)	5 (25%)	30 (58.8%)	21 (41.2%)	6 (28.6%)	15 (71.4%)	17(44.7%)	21 (55.3%)	.01
	*Recommended treatment in PC*	11 (55%)	9 (45%)	36 (70.6%)	15 (29.4%)	18 (85.7%)	3 (14.3%)	26 (68.4%)	12 (31.6%)	.19
	*Antidepressant prescription*	15 (75%)	5 (25%)	29 (56.9%)	22 (43.1%)	8 (38.1%)	13 (61.9%)	27 (71.1%)	11 (28.9%)	.04

Table 3 shows the associations of demographic (age, gender, country) and professional variables (years of training) with the clinicians’ recognition of and treatment decisions regarding common mental health problems using the GLM. In the depression vignette, nationality, gender, age, and years of training had an association with clinical decisions. The psychiatrists from Brazil were less likely to recognize a mental disorder than the professionals from Cuba (odds ratio (OR) = 0.30, 95% CI, 0.10 to 0.91, *p* < 0.04), and females were less likely to consider treating these patients in PC than males (OR = 0.19, 95% CI, 0.04 to 0.91, *p* < 0.02). At the same time, older age was associated with a lower likelihood of agreement that treatment by a PCP was appropriate (OR = 0.92, 95% CI, 0.87 to 0.97, *p* < 0.01) and suggesting an antidepressant prescription (OR = 0.94, 95% CI, 0.89 to 0.84, *p* < 0.01). Also, more years of training increased the chances of antidepressant prescription (OR = 1.15, 95% CI, 1.05 to 1.25, *p* < 0.01). In the somatoform symptoms vignette, training years in the mental health area was correlated with clinical decisions. More years of training increased the probability of choosing treatment by psychiatrists over PCPs (OR = 1.19, 95% CI, 1.04 to 1.37, *p* < 0.01). In the anxiety vignette, participants from Bolivia were more likely to prefer treatment by psychiatrists over PCPs than those from Cuba (OR = 4.19, 95% CI, 1.22 to 14.42, *p* < 0.02), and being female increased the probability of these patients being treated by psychiatrists in comparison with males (OR = 2.38, 95% CI, 1.10 to 5.13, *p* < 0.01). The professionals from Chile were less likely than those from Cuba to prescribe antidepressants (OR = 0.29, 95% CI, 0.08 to 0.99, *p* < 0.05).

**Table 3:** Associations of demographic/professional variables with clinicians’ recognition of mental disorders and treatment decisions using the generalized linear model.

Model*						
	Depression		Somatoform		Anxiety	
	OR (95%)	p-value	OR (95%)	p-value	OR (95%)	p-value
*Recognized a mental disorder*					-	-
Nationality (Bolivia)	0.86(7.39–5.47)	0.87	0.68(0.14–1.30)	0.63	-	-
Nationality (Chile)	3.42(0.37–31.9)	0.28	0.81(0.14–4.70)	0.82	-	-
Nationality (Brazil)	**0.30 (0.10–0.91)**	**0.04**	0.47(0.15–1.49)	0.20	-	-
Gender (Female)	1.24 (0.54–2.87)	0.62	0.53 (0.22–1.27)	0.15	-	-
Age	1.02 (0.96–1.07)	0.58	1.02 (0.95–1.08)	0.63	-	-
Years of training	0.93 (0.86–1.01)	0.08	0.98 (0.90–1.07)	0.60	-	-
*Recommended psychiatric treatment*						
Nationality (Bolivia)	0.88(5.36–3.89)	0.85	22.9(21.9–23.9)	0.09	**4.19 (1.22–14.42)**	**0.02**
Nationality (Chile)	0.45(0.14–1.47)	0.19	0.56(0.12–0.39)	0.46	0.42(0.12–1.48)	0.18
Nationality (Brazil)	1.61(2.78–3.97)	0.30	**6.07 (2.37–15.51)**	**0.01**	1.70(0.70–4.10)	0.24
Gender (Female)	1.46 (0.69–3.08)	0.33	0.79 (0.32–1.94)	0.61	**2.38 (1.10–5.13)**	**0.03**
Age	1.03 (0.99–1.07)	0.10	0.97 (0.92–1.03)	0.33	0.98 (0.94–1.02)	0.26
Years of training	1.03 (0.94–1.12)	0.54	**1.19 (1.04–1.37)**	**0.01**	1.05 (0.98–1.12)	0.14
*Willing to allow treatment in PC*						
Nationality (Bolivia)	**20.2 (18.4–22.5)**	**0.01**	0.78(0.17–3.69)	0.75	0.54(0.16–1.80)	0.32
Nationality (Chile)	0.82(0.14–4.75)	0.83	**0.27 (0.34–1.88)**	**0.01**	2.51(0.60–10.5)	0.21
Nationality (Brazil)	0.43(0.08–2.44)	0.34	0.27(0.10–0.72)	**0.13**	1.07(0.42–2.72)	0.90
Gender (Female)	**0.19 (0.04–0.79)**	**0.02**	0.80 (0.10–0.72)	0.60	1.10 (0.50–2.44)	0.81
Age	**0.92 (0.87–0.97)**	**0.01**	1.04 (0.96–1.11)	0.34	1.02 (0.98–1.07)	0.24
Years of training	1.01 (0.91–1.04)	0.84	**0.88 (0.80–0.96)**	**0.01**	0.99 (0.93–1.07)	0.94
*Antidepressant prescription*						
Nationality (Bolivia)	**22.26 (21.09–23.44)**	**0.01**	18.1(15.6–20.6)	0.08	1.59(0.39–6.53)	0.52
Nationality (Chile)	1.31(0.39–4.43)	0.66	17.7(15.1–20.4)	0.09	**0.29 (0.08–0.99)**	**0.05**
Nationality (Brazil)	2.82(0.86–9.21)	0.09	0.22(0.01–3.35)	0.28	0.53(0.18–1.54)	0.24
Gender (Female)	1.07 (0.43–2.66)	0.88	2.72 (0.08–89.65)	0.57	0.62 (0.27–1.38)	0.24
Age	**0.94 (0.89–0.84)**	**0.01**	1.38 (0.90–2.12)	0.14	**0.91 (0.87–0.95)**	**0.01**
Years of training	**1.15 (1.05–1.25)**	**0.01**	0.90 (0.77–1.05)	0.19	1.02 (0.95–1.09)	0.66

*Logistics regressions where the dependent variables (outcome) were the chances of “yes” responses (recognizing a disease, willing to allow treatment, referring, and antidepressant prescription) compared with “no.” Abbreviations: CI = confidence interval; p = p value.

** Country of reference in the model: Cuba.

## Discussion

In our study we explore clinical decisions of psychiatrists regarding the management of common mental disorders in primary care (PC) in four Latin American countries, applying a questionnaire and clinical vignettes. Despite important differences in each country in contexts, infrastructure in the health area, quantity of professionals, medicaments used, and policies concerning the treatment of mental disorders, in our sample, most of the psychiatrists considered that common mental disorders presented in the vignettes, could in principle, be treated by PCPs. Also, gender, age, experience, and years of training are related to psychiatrists’ perceptions of whether they should treat common mental disorders or whether PCPs should treat these patients in PC settings.

The four countries of our sample have important differences in their mental health policies; for example Cuba and Chile have higher levels of development and organization of PC systems than do Bolivia and Brazil [8, 14, 19, 20, 25], and another important difference was the number of psychiatrists per 100,000 inhabitants, with Cuba having the highest rate of 11 and Bolivia the lowest of 1.30 [21, 22, 26]. In those countries with a better level of organization and development of primary care services, and with a greater number of psychiatrists, primary care professionals could have a better level of knowledge to treat patients with common mental disorders.

Another important difference that influenced the clinical decisions of the psychiatrists in those countries was that Cuban and Chilean psychiatrists had more training and more professional experience working in PC settings than psychiatrists from Bolivia and Brazil. The findings of this study show that most of the psychiatrists from the four countries considered that patients with depression could be treated by PCPs, though indicating that patients with anxiety and somatoform symptoms should be referred to specialized care.

After adjusting for the association of sociodemographic variables and countries with clinicians’ recognition of common mental disorders and their subsequent treatment decisions, we found that in the case of somatoform symptoms, more training was linked to the decision to opt for specialized care as opposed to PC. In the case of anxiety symptoms, psychiatrists from Bolivia were more likely to consider psychiatric treatment than professionals from Cuba, and females were more likely to choose psychiatric treatment over PC. In a study that used these same vignettes in a sample of PCPs from these four countries, we found that the majority of PCPs considered themselves prepared to treat these patients, but only the PCPs from Brazil were more likely to decide to refer patients with somatoform symptoms to specialized care [22].

The possible factors that influence the referral from PCPs to mental health specialists, particularly in the case of depression, are well described in the literature: patient-related factors, clinician-related issues, practice environment-related issues, and perceived severity of symptoms. Other factors include patient attitudes, characteristics, access to mental health services, and clinicians’ experience [27–37]. In addition, some studies have suggested that a lack of knowledge and a lack of training and experience increase clinicians’ levels of discomfort when working with patients with mental disorders [38–43]. Also, increased knowledge in a particular clinical domain usually increases referrals, possibly because more knowledgeable PCPs are more attuned to clinical complexities [43–45].

However, the perceptions of psychiatrists and the possible factors that influence their judgment regarding the treatment of common mental disorders in PC have not received much attention. It is possible that psychiatrists consider PCPs unprepared to manage common mental disorders. Lower levels of knowledge, lack of adequate training, and stigmatizing attitudes have been associated with poorer diagnostic and treatment efficacy in mental disorders in PC [46–51]. On the other hand, recent studies report a lack of collaboration [52, 53] and consensus on protocol of care [54, 55] between PCPs and psychiatrists, and some psychiatrists perceived a lack of time and remuneration to sustain their regular visits to primary care settings [17].

Professionals from Chile and Cuba are more prepared to see patients in PC because the PC systems have programs in their policies to support mental health treatment of common mental disorders such as depression and anxiety in PC by a group of trained PCPs. Moreover, they consist of psychologists, nurses, and social workers, which gives psychiatrists greater confidence of maintaining the treatment of these patients in PC. In other studies, PCPs and psychiatrists have supported the management of common mental health problems in PC, but support from private psychiatrists has been significantly lower [18]. In developed countries, PCPs are the point of entry into the health care system and the most frequently consulted professionals for mental health problems, followed by mental health specialists (for example, psychiatrists) [56, 57]. However, PCPs have the lowest rate of providing adequate treatment for common mental health disorders. Depending on the type of health care system, PCPs may or may not collaborate closely with mental health professionals, especially with psychiatrists. Most practice guidelines recommend gatekeeping arrangements between PCPs and mental health professionals, or at least facilitating collaboration among professionals in order to offer adequate care to as many patients as possible [56]. The WHO recommendation is that low- and medium-complexity disorders can and should be treated in PC settings, not only because of a lack of psychiatrists but also because it would facilitate timely treatment in the context in which people live, with due training of the PCPs in psychosocial, psycho-educational, and medical tools as appropriate, thus reducing the burden of disorders and the associated stigma [25].

This study has several limitations. First, the sample was limited to professionals from the four countries of interest who had access to the internet, except that in Cuba we applied the questionnaire in paper form; therefore, they may not be considered representative of all psychiatrists from their countries because we applied the questionnaires in specific cities and used convenience sampling. Second, a convenience sample was used, which limits the generalizability of the results. Third, the vignettes used in this study were based on written cases presenting symptoms of three common mental disorders, which may not be representative of live interactions with a patient; and third, our study only examined the associations between gender, attitudes, and years of training and experience, and did not measure other potential predictors in relation to the management of common mental disorders by the psychiatrists in PC settings. Despite these limitations, these findings constitute an important step in understanding the interaction between PCPs and psychiatrists in the management of common mental disorders in PC in Latin American countries.

The main conclusions of this study are that gender, age, experience, and years of training are related to psychiatrists’ perceptions of whether they should treat common mental disorders or whether PCPs should treat these patients in PC settings. Most of the psychiatrists considered that patients with depression symptoms could be treated by PCPs, while there were country-based differences regarding patients with somatoform and anxiety symptoms. It is necessary to develop protocols and collaboration models between psychiatrists and primary care professionals, in addition to favoring the training of primary care professionals for the identification and treatment of common mental disorders.

## Supporting information

S1 File Questionnaire_primary care English. (PDF)

S2 File Questionnaire_primary care Spanish. (PDF)

S3 File Questionnaire_primary care Portuguese. (PDF)
