# Supplementary material for: Management of common mental disorders should take place in primary health or specialized care? Clinical decisions of psychiatrists from Latin American countries
Source: PLoS One. 2022 Apr 5;17(4):e0265308. doi: 10.1371/journal.pone.0265308 (PMC8982860; doi:10.1371/journal.pone.0265308)
Supplement: S1 File — (PDF) [file pone.0265308.s001.pdf]

Primary Care Physicians Spanish

Q1 Dear colleague, we invite you to participate in this study on issues related to the treatment of mental disorders in primary care (basic), all the information provided will have maximum confidentiality, contributing to the development of three lines of research that are being developed at the Federal University of São Paulo (UNIFESP), Department of Psychiatry, in 4 countries: Brazil, Bolivia, Chile, Cuba.

Q2 I am a Primary Care Physician qualified to practice in my country, and I agree to provide information regarding the questions asked in this questionnaire which will help improve the care of patients with mental disorders in Primary Health Care.
❍ Yes (1)
❍ No (2)

Q3 Do you currently see patients as part of your regular professional activities?

❍ Yes (1)
❍ No (2)

Q4 Gender
❍ Female (1)
❍ Male (2)

Q5 Nationality

Q6 In what city do you work?

Q7 Year of Birth

Q8 What languages do you most frequently use in your professional life?

❑ English (1)
❑ Portuguese (2)
❑ Spanish (3)

Q9 What is your clinical profession?

Q10 How many years of formal professional training you have, not including the university degree? (Note that we are referring only to years of formal training, not to years of experience. Formal training includes post-university graduate education, professional and specialty training such as internships, residencies, and postdoctoral fellowships, but does not include routine continuing professional education).

Q11 As part of your professional training (academic and clinical), which of the following diagnostic systems for mental and behavioral disorders were you trained to use? (Check all that apply):

- ICD-10

- ICD-9
- ICD-8
- DSM-IV
- DSM-III-R
- DSM-III
- None
- Other; Please specify:

Q12 What are your primary work settings at the present time? By primary work setting, we mean one where you work for about five hours or more per week. (Check all that apply.)

- General primary care setting
- Other general medical setting (including university medical center, medical school)
- Mental health services setting (including university-based facility, hospital department, outpatient clinic)
- Substance abuse specialty program
- Private clinical practice (solo or group)
- University (not in medical, mental health, or substance abuse treatment setting)
- Public health administration or government agency
- Non-governamental organization (NGO)

Q13 Years of professional experience in your field (after completing your formal training): ____.

Q14 Please indicate which of the following types of mental health services you personally provide to patients as part of your regular professional activities. (Check all that apply)

- Diagnostic assessment of mental and behavioral disorders
- Evaluation and management of psychoactive medications
- Psychological assessment (e.g., neuropsychological evaluation, personality testing, etc.)
- Psychological (talk) therapy
- Psychoeducation

- Other (specify):
- None

Q15 Do you directly supervise the provision of health services by others? (By direct supervision, we mean that you monitor the services provided through such mechanisms as face-to-face supervision, case conferences, chart review, and that you are directly accountable for the quality of the clinical services that they provide.)

- Yes (1)
- No (2)

Q16 How many people do you supervise directly in their provision of health services?

Q17 Please indicate which of the following types of mental health services are provided by the people that you supervise. (Check all that apply.)

- Diagnostic assessment of mental and behavioral disorders
- Evaluation and management of psychoactive medications
- Psychological assessment (e.g., neuropsychological evaluation, personality testing, etc.)
- Psychological (talk) therapy
- Psychoeducation
- Other (specify):
- None

Q18 During a typical week, in what types of settings do you provide or supervise health services? (Check all that apply)

Mental health services settings:

- Hospital
- Primary care center
- Domiciliary
- Partial hospitalization/Day hospital
- Private Clinic
- Private practice

Q19 During a typical week, in what types of settings do you provide or supervise health services? (Check all that apply) Substance abuse specialty program:

- Hospital

- Primary care center
- Domiciliary
- Partial hospitalization/Day hospital
- Private Clinic
- Private practice

Q20 During a typical week, in what types of settings do you provide or supervise health services? (Check all that apply) General medical settings:

- Hospital
- Primary care center
- Domiciliary
- Partial hospitalization/Day hospital
- Private Clinic
- Private practice

Q21 During a typical week, in what types of settings do you provide or supervise health services? (Check all that apply) Other settings:

- Hospital
- Primary care center
- Domiciliary
- Partial hospitalization/Day hospital
- Private Clinic
- Private practice

Q22 In what type of community are the service settings where you provide or supervise health services located? (If you provide services in more than one setting, check all that apply.)

- Major urban Center
- Suburb of a major urban center
- Mid-size city
- Smaller city or town
- Village

- o Rural setting

Q23 For the health services that you provide or supervise, what are the percentages in each patient age group? (Percentages should total 100.)

- o Children (0-12): ____.
- o Adolescents (13-18): ____.
- o Adults (18-65): ____.
- o Elderly (65 and over): ____.
- o Total: 100 %.

Q24 Who is most often responsible for assigning a psychiatric diagnosis to the patients whose services you provide or supervise?

- o I or someone under my direct supervision usually assigns the diagnosis.
- o Another health professional not under my supervision usually assigns the diagnosis (e.g., an attending physician).
- o Diagnoses are usually assigned by medical records coders.
- o Psychiatric diagnoses are usually not assigned.
- o Other (specify):

Q25 Please indicate how regularly you use the following classification systems in your clinical practice and/or supervision:

ICD-10:

- o Routinely
- o Often
- o Sometimes
- o Rarely
- o Never

ICD-9 or ICD-9-CM:

- o Routinely
- o Often
- o Sometimes
- o Rarely

- Never

DSM-IV or DSM-IV-TR:

- Routinely
- Often
- Sometimes
- Rarely
- Never

Other (specify):

- Routinely
- Often
- Sometimes
- Rarely
- Never

Q26 Based on the classification system that you use most frequently in your clinical practice, do you think that classification makes it easier to diagnose patients with mental health problems in primary care?

❍ Yes (1)
❍ No (2)

Q27 The classification system that you use most frequently in relation to mental health disorders, does it adjust to the symptomatology that your patients present in your clinical practice in primary care?

❍ Yes (1)
❍ No (2)

Q28 Do you have administrative responsibility for one or more service units that provide mental health services, even if you do not personally provide direct clinical care or supervision?

❍ Yes (1)
❍ No (2)

Q29 How many people provide mental health services in the units for which you have responsibility? Number of people: ____.

Q30 During a **typical week**, how many **hours** do you devote to each of the following professional activities?

- Providing direct mental health services to patients (e.g., assessment, psychological or behavioral therapies, medication management)

- Providing other health care services (not mental health)
- Supervision of health services provided by others:
- Teaching
- Research
- Administration
- Other (specify):

Q31 Please select up to three areas related to the ICD-10 Mental and Behavioural disorders in which you have the most knowledge and experience.

- Dementia, Delirium, and Related Disorders
- Substance-Related Disorders and Behavioural Addictions
- Schizophrenia and Related Disorders
- Mood Disorders
- Anxiety Disorders
- Stress-Related Disorders
- Obsessive-Compulsive and Related Disorders
- Somatoform Disorders
- Eating Disorders
- Sleep Disorders
- Sexual Disorders
- Personality Disorders
- Intellectual Disabilities
- Autism Sprectrum Disorders
- Attention Deficit and Conduct Disorders
- Epidemiology
- Public Health
- Neuroscience
- Other (specify):

Q32 I feel sufficiently trained to diagnose and treat patients with common mental disorders:

- ❍ I fully agree (1)
- ❍ Partially Agree (2)
- ❍ Disagree Partially (3)
- ❍ Desacuerdo Plenamente (4)

Q33 Please provide any additional comments about your work settings, interests or expertise that you feel have not been adequately addressed in this questionnaire:

Q34 Please answer the questions as follows according to your clinical decision for the case:

Referral:

MDS, female, 53 years old, came to the Health Unit complaining about dizziness.

Presenting Symptoms:

MDS is diabetic with a good glycaemia control. She denies nausea, dry mouth, abdominal pain or urinary symptoms. She says that her dizziness started a day before, but it is getting worse. The nurse asks her if she had some trouble and she started to cry, saying that she has frequently being with dizziness, especially when she has to deal with problems with her son.

Additional Background Information:

Since her only son started to “do bad things because of friends”, about a year ago, she doesn’t want to go out of the bed. She cries many times a day and beliefs to be a sad person. She is not eating well because feels fullness and nausea, and she complains of recurrent nightmares. The clinical exam shows the patient is hydrated, with no signs of any problem in abdominal and neurological scrutiny. Her blood pressure is 140x90mmHg and her peripheral glycaemia is 248mg/dL.

	Strongly agree	Somewhat agree	Somewhat disagree	Strongly disagree
**33.1. The patient should be treated for complication of acute diabetes.**	☐	☐	☐	☐
**33.2. You should be given a benzodiazepine and can be sent for their home.**	☐	☐	☐	☐
**33.3. MDS can have a major mental disorder.**	☐	☐	☐	☐
**33.4. General / Family Physician in the community should treat diabetes and refer the patient to a [illegible]**	☐	☐	☐	☐

**33.5. MDS provides a picture of depression that should, in principle, be handled by the General Medical / Family.**				
**33.6. MDS has a symptoms of anxiety that needs treatment.**				
**33.7. MDS has a major depression Medical General / Family should be treated with an antidepressant.**				

2. Please answer the questions as follows according to your clinical decision for the case:

Referral:

LCP, a 54-year-old woman, came for consultation complaining about pain in her arms.

Presenting Symptoms:

The patient is complaining to present pain for 6 years, sometimes worsening, besides formicating crisis in the right side of the body, during 2 to 4 hours each one, with a frequency of 2 times a week. She also refers excessive tiredness and gastric bloating. She brings complementary exams to the consultation done in different health services in the last year: cell blood counting, glycaemia, lypemia, electrolytes measures, hepatic enzymes, thyroid hormones, endoscopy, cranium and column tomography, Doppler ultrasonography of carotids and vertebral arteries and electromyography, all of them with no apparent abnormalities.

Additional Background Information:

She has been unable to work in the last 4 years, since the death of her son. She says not enjoy going out of home, she avoids meeting friends and relatives and has sex with her husband “for obligation”. She notices that the symptoms are much worse when she feels nervous. She is taking non-steroids anti-inflammatory analgesics and muscle relaxant drugs very often, actually with no improvement. She denies presenting a chronic disease. In the consultation, her blood pressure is 120x75mmHg; she has pain in muscle palpation of trapezium and rhomboid, without limitation of movements. The neurological examination shows normal strength, tonus, reflexes, proprioception and coordination.

	Strongly agree	Somewhat agree	Somewhat disagree	Strongly disagree

**34.1. The patient should be given a more profound clinical study to identify a physical illness**				
**34.2. It should be administered for the patient a benzodiazepine for continuous use**				
**34.3. MDS can have a major mental disorder.**				
**34.4. LCP would benefit from the use of antidepressant.**				
**34.5. General / Family Physician must refer the patient for a psychiatrist.**				
**34.6. LCP has a mental disorder should, in principle, be handled by the General Medical / Family.**				
**34.7. LCP no organic disease therefore does not require treatment.**				

3. Please answer the questions as follows according to your clinical decision for the case:

Referral:

ACV, male, 46 years old, comes to the health unit with chest pain.

Presenting Symptoms:

He refers tightness chest pain in the left side of the thorax, not associated with physical effort, and started 4 months ago, getting more frequently since the beginning of this symptom. Eventually, the pain is associated with dyspnea, sometime with paresthesia in both hands. He says that sometimes presents shakes and dizziness. When asked about the symptoms, he attributes the symptoms to a possible restless sleep. He feels angry and often agues with his wife, but doesn't believe that these symptoms has anything to do with his emotions.

Additional Background Information:

The patient tells he has a 24-year-old son that will get marry in a month and his 19-year-old daughter wants to go to university, but he is not sure to be able to afford her studies. He is hypertensive, taking Hydroclorotiazide 25mg in the morning and Enalapril 5mg every 12 hours. He denies family history of cardiovascular disease. He is a tradesman, working 12 hours a day. Not smoker. He has additional exams done last month: glycaemia 99mg/dL, total cholesterol 223 mg/dL, HDL 65mg/dL, triglycerides 145mg/dL, creatinine 0,9mg/dL, potassium 3,8mmol/L, no proteinuria, and electrocardiogram with sinus rhythm with no abnormalities.

	Strongly agree	Somewhat agree	Somewhat disagree	Strongly disagree
**35.1. The patient only needs a study for a coronary syndrome.**	☐	☐	☐	☐
**35.2. Is advised to the patient the antidepressant use.**	☐	☐	☐	☐
**35.3. ACV has indicated the use of a benzodiazepine.**	☐	☐	☐	☐
**35.4. General / Family Physician must refer the patient for a psychiatrist.**	☐	☐	☐	☐
**35.5. ACV presents a picture of anxiety that should, in principle, be treated by the Medical General / Family.**	☐	☐	☐	☐
**35.6. ACV does not require any current psychiatric treatment only for hypertension.**	☐	☐	☐	☐

Mental Illness: Clinicians' Attitudes Scale MICA-4

Note to researchers distributing this scale: please only use after reading instructions in "Manual for Researchers".

Instructions: for each of questions 1-16, please respond by **ticking one box only**. Mental illness here refers to conditions for which an individual would be seen by a psychiatrist.

		Strongly agree	Agree	Somewhat agree	Somewhat disagree	Disagree	Strongly disagree
1	I just learn about mental health when I have to, and would not bother reading additional material on it.						
2	People with a severe mental illness can never recover enough to have a good quality of life.						
3	Working in the mental health field is just as respectable as other fields of health and social care.						
4	If I had a mental illness, I would never admit this to my **friends** because I would fear being treated differently.						
5	People with a severe mental illness are dangerous more often than not.						
6	Health/social care staff know more about the lives of people treated for a mental illness than do family members or friends.						
7	If I had a mental illness, I would never admit this to my **colleagues** for fear of being treated differently.						
8	Being a health/social care professional in the area of mental health is **not** like being a real health/social care professional.						
9	If a senior colleague instructed me to treat people with a mental illness in a disrespectful manner, I would **not** follow their instructions.						

Mental Illness: Clinicians' Attitudes Scale MICA-2 © 2010. Health Service and Population Research Department, Institute of Psychiatry, King's College London.
We would like to thank Aliya Kassam for her major contribution to the development of this scale.
Contact: Professor Graham Thornicroft. Email: graham.thornicroft@kcl.ac.uk

Kassam A., Glozier N., Leese M., Henderson C., Thornicroft G. (2010) Development and responsiveness of a scale to measure clinicians' attitudes to people with mental illness (medical student version). Acta Psychiatrica Scandinavica 122(2), 153-161.

Mental Illness: Clinicians' Attitudes Scale

MICA-4

Note to researchers distributing this scale: please only use after reading instructions in "Manual for Researchers".

Instructions: for each of questions 1-16, please respond by **ticking one box only**. Mental illness here refers to conditions for which an individual would be seen by a psychiatrist.

		Strongly agree	Agree	Somewhat agree	Somewhat disagree	Disagree	Strongly disagree
10	I feel as comfortable talking to a person with a mental illness as I do talking to a person with a physical illness.						
11	It is important that any health/social care professional supporting a person with a mental illness also ensures that their physical health is assessed.						
12	The public does **not** need to be protected from people with a severe mental illness.						
13	If a person with a mental illness complained of physical symptoms (such as chest pain) I would attribute it to their mental illness.						
14	General practitioners should **not** be expected to complete a thorough assessment for people with psychiatric symptoms because they can be referred to a psychiatrist.						
15	I would use the terms 'crazy', 'nutter', 'mad' etc. to describe to colleagues people with a mental illness who I have seen in my work.						
16	If a colleague told me they had a mental illness, I would still want to work with them.						

Thank you very much for your help.

Mental Illness: Clinicians' Attitudes Scale MICA-2 © 2010. Health Service and Population Research Department, Institute of Psychiatry, King's College London.
We would like to thank Aliya Kassam for her major contribution to the development of this scale.
Contact: Professor Graham Thornicroft. Email: graham.thornicroft@kcl.ac.uk

Kassam A., Glozier N., Leese M., Henderson C., Thornicroft G. (2010) Development and responsiveness of a scale to measure clinicians' attitudes to people with mental illness (medical student version). Acta Psychiatrica Scandinavica 122(2), 153-161.
